# Correlations among workplace bullying, work stress, and social support: a meta-analysis with structural equation modeling

**DOI:** 10.3389/fpsyg.2026.1817200

**Published:** 2026-05-08

**Authors:** Yin-Che Chen, Chen-Ling Chen, Hui-Chuang Chu

**Affiliations:** Department of Educational Psychology and Counseling, National Tsing Hua University, Hsinchu, Taiwan

**Keywords:** meta-analysis, social support, structural equation modeling, work stress, workplace bullying

## Abstract

**Introduction:**

Workplace bullying and work stress not only harm individuals but also adversely affect organizations as a whole. Strengthening social support may mitigate the negative effects of work stress and enhance overall job performance. Existing research has largely focused on relationships between individual variables, with relatively limited attention given to their integrated interactions, particularly in the domestic context, where studies on workplace bullying, work stress, and social support remain insufficient.

**Methods:**

This study employed meta analysis and structural equation modeling to examine the interrelationships among workplace bullying, work stress, and social support, with the aim of providing more systematically grounded findings and proposing effective response strategies.

**Results:**

The results indicated a moderate positive correlation between workplace bullying and work stress, r = 0.517, a small negative correlation between workplace bullying and social support, r = 0.210, and a small negative correlation between work stress and social support, r = 0.201. In the observed variable path analysis based on the pooled correlation matrix, workplace bullying was associated with work stress both directly, *β* = 0.497, and indirectly through reduced social support, *β* = 0.020, yielding a total effect of *β* = 0.517. The indirect pathway was modest relative to the direct pathway, indicating that social support functions as a supplementary mechanism in the association between workplace bullying and work stress.

**Discussion:**

These findings indicate that social support partially accounts for the relationship between workplace bullying and work stress, while the direct pathway remains the predominant channel through which bullying elevates stress. Based on these findings, this study recommends that organizations cultivate a positive work environment, provide psychological support, workplace counseling, and training, strengthen anti bullying policies, and enhance employees’ resilience and stress management skills in order to improve job performance.

## 1 Research background and motivation

Workplace bullying has become a major concern in contemporary organizations because of its harmful consequences for both employees and organizational functioning. Such experiences often evoke feelings of defeat, threat, humiliation, and isolation, thereby undermining self-confidence and is associated with substantial psychological and physical strain (Taris, 2022). International meta-analytic evidence has consistently confirmed the detrimental effects of workplace bullying. Nielsen and Einarsen (2012) reported robust associations between bullying exposure and mental health problems, psychosomatic complaints, and diminished well-being across diverse occupational samples, whereas Verkuil et al. (2015) demonstrated that bullying is associated with physiological stress indicators, including elevated cortisol levels and cardiovascular reactivity. At the organizational level, workplace bullying undermines employee morale, increases turnover, damages organizational reputation, disrupts normal workplace operations, and compromises work performance and quality of life (Chang et al., 2018; Hoel et al., 2020; Li, 2018; Tai, 2020).

Work stress likewise remains a central issue in organizational research and practice. Excessive work stress is associated with considerable health and financial costs on employees and organizations alike (Karatepe et al., 2018). Although moderate stress may stimulate individual potential and enhance work efficiency, excessively high or low stress can reduce effectiveness and adversely affect both employees and their work environments (Bottiani et al., 2019). When work demands are perceived as overwhelming, individuals may develop physical and psychological difficulties, underscoring the importance of organizational support and resources in stress management (He et al., 2020). Research on the antecedents of bullying has further shown that adverse work environment conditions, including role conflict, role ambiguity, and high workload, are associated with both the prevalence of bullying and the severity of work stress (Bowling and Beehr, 2006; Hauge et al., 2010).

Social support is widely recognized as a key protective resource in workplace settings. In demanding work environments characterized by high efficiency expectations and long working hours, adequate social support is associated with lower work stress, strengthen coping capacity, improve performance, and enhance willingness to remain with the organization (Chiu, 2021). Previous studies have further shown that social support can directly reduce perceived stress and improve physical and mental health (Hwang, 2010; Wu, 2017). In addition, social support exerts a buffering function whereby support from family members, friends, colleagues, and supervisors may alleviate stress and reduce its detrimental effects (Nielsen et al., 2020; Yeh and Tseng, 2022).

Despite the importance of workplace bullying, work stress, and social support, prior research has tended to focus on bivariate relationships rather than on their integrated interplay (Samnani and Singh, 2012). This limitation is especially notable in the domestic literature, where studies jointly examining these three constructs remain relatively limited. Existing research has largely relied on quantitative survey methods conducted within specific occupational groups, including nursing (Alshawush et al., 2022; Feng et al., 2020), education (Fahie, 2014), and hospitality (Karatepe et al., 2018). Given the accumulation of empirical studies across Taiwan and East Asia, an integrated examination of these variables through meta analysis and structural equation modeling provides an appropriate basis for clarifying their interrelationships within this cultural context and for developing a more systematic theoretical account of how they jointly operate in East Asian organizational settings.

The geographic focus on Taiwan and East Asia is deliberate and theoretically motivated. Organizational environments in these societies are characterized by relatively high power distance, collectivist interpersonal norms, Confucian heritage values emphasizing hierarchical harmony and face preservation, and institutional structures that shape both the expression of workplace bullying and the mobilization of social support in ways that may differ substantively from Western contexts (Hofstede, 2001; Kim et al., 2008; Loh et al., 2010). By restricting the evidence base to a culturally coherent region, the present study avoids conflating culturally heterogeneous effect sizes and instead provides a regionally grounded basis for testing the proposed explanatory model. The findings are therefore interpretable as evidence of the correlational structure among workplace bullying, work stress, and social support as it operates within East Asian organizational norms, thereby providing a necessary empirical foundation for subsequent cross cultural comparison.

The conceptual model of the present study is grounded in three complementary theoretical perspectives: the Job Demands-Resources model (Bakker and Demerouti, 2007; Demerouti et al., 2001), Conservation of Resources theory (Hobfoll, 1989, 2001), and the Transactional Model of Stress (Lazarus and Folkman, 1984). The Job Demands-Resources model proposes that all work characteristics can be classified as either job demands or job resources. Job demands are physical, psychological, organizational, or social aspects of work that require sustained effort and are associated with physiological and psychological costs, whereas job resources are aspects of work that facilitate goal attainment, reduce job demands, and stimulate personal growth. Within this framework, workplace bullying is conceptualized as a social job demand because repeated exposure to hostile, humiliating, or intimidating behaviors imposes sustained psychological costs on targeted employees and activates a health impairment process that manifests as elevated work stress, emotional exhaustion, and burnout (Bakker and Demerouti, 2007). Social support, in turn, is conceptualized as a job resource that operates through both direct and buffering pathways. As a direct resource, support from supervisors and coworkers facilitates goal attainment and reduces the perceived severity of workplace demands, whereas as a buffer it attenuates the adverse effects of demands such as bullying on strain outcomes (Bakker et al., 2005).

Conservation of Resources theory provides a complementary lens by focusing on the role of resource dynamics in the stress process (Hobfoll, 1989, 2001). According to this perspective, individuals strive to obtain, retain, and protect valued resources, including objects, conditions, personal characteristics, and social relationships, and they experience stress when these resources are threatened, lost, or inadequately replenished. Workplace bullying constitutes a potent resource threat because it erodes interpersonal relationships, diminishes self-efficacy, and undermines the sense of control and belonging that employees derive from their work environment. As bullying depletes social resources, employees become increasingly vulnerable to further resource loss in what Hobfoll (2001) termed a resource loss spiral. Social support functions as a critical resource reservoir that may interrupt this spiral. However, because bullying often targets and erodes interpersonal bonds, the availability of social support may itself be diminished as a consequence of bullying, thereby limiting its protective capacity.

Lazarus and Folkman’s (1984) Transactional Model of Stress further provides a process level explanation of how individuals appraise and respond to workplace stressors. This model distinguishes between primary appraisal, in which an event is evaluated as irrelevant, benign positive, or stressful, and secondary appraisal, in which available coping resources are assessed. Workplace bullying, by virtue of its repeated and interpersonally hostile nature, is likely to be appraised as a threat or harm/loss event, thereby triggering stress responses. The availability of social support influences secondary appraisal because individuals who perceive adequate support from colleagues, supervisors, or family members are more likely to regard their coping resources as sufficient, thereby reducing the perceived stressfulness of the bullying experience. This model therefore clarifies the micro level cognitive mechanism through which social support may partially mediate the relationship between workplace bullying and work stress: bullying that erodes social support reduces the coping resources available for secondary appraisal and indirectly amplifies the stress response beyond the direct impact of the bullying behavior itself (Lazarus and Folkman, 1984).

Integrating these three perspectives, the present study positions workplace bullying as a salient social job demand within the Job Demands-Resources framework, as a resource threat within Conservation of Resources theory, and as a stressor appraised as harmful or threatening within the Transactional Model of Stress. Workplace bullying may directly elevate work stress through a health impairment process and resource depletion, while simultaneously diminishing the social support available to employees. Social support is conceptualized as a job resource, a valued interpersonal resource, and a coping resource that shapes secondary appraisal. Within this framework, the interrelationships among workplace bullying, work stress, and social support are not independent pairwise associations. Rather, they constitute an integrated explanatory structure in which social support may account for part of the relationship between workplace bullying and work stress through a resource depletion and impaired appraisal pathway.

A substantial body of international research has demonstrated the adverse effects of workplace bullying on individuals and organizations. Bullying is widely recognized as an extreme social stressor that is associated with anxiety, helplessness, emotional exhaustion, and turnover tendencies (Alshawush et al., 2022; Chang, 2022). Nielsen and Einarsen (2012), in their comprehensive meta analysis of 66 studies, reported that workplace bullying was associated with a wide range of negative outcomes, including mental health problems, physical health complaints, posttraumatic stress symptoms, and diminished job satisfaction. These findings have been corroborated by Verkuil et al. (2015), who extended the evidence to physiological outcomes, demonstrating associations between bullying and cortisol dysregulation, cardiovascular reactivity, and sleep disturbances. At the organizational level, bullying is associated with poorer workplace climates, negative attitudes and behaviors, and increases fatigue, irritability, and alienation (Feijó et al., 2019; Kim et al., 2019). Bowling and Beehr (2006), in a meta analysis of workplace harassment, further showed that harassing experiences were substantially associated with indicators of strain, negative emotions, and reduced organizational commitment across diverse industry samples.

The work environment conditions under which bullying occurs are also well documented. Hauge et al. (2010) identified role conflict, role ambiguity, and high workload as key predictors of bullying in a large Norwegian sample. In the Taiwanese context, Feng et al. (2020) argued that bullying and stress are latent turnover factors among nurses. Lee and Jung (2020) showed that staff relationships, supervisor management, and institutional support affect bullying severity in small and medium hospitals. These patterns extend beyond healthcare. In hospitality, employees face high service standards, unclear tasks, and managerial pressure that function as precursors to bullying and work stress (Karatepe et al., 2018; Liu and Chou, 2016). In education, Fahie (2014) documented comparable dynamics among school staff. Within the Job Demands-Resources framework, these conditions represent elevated job demands that, in the absence of adequate resources, initiate the health impairment process linking bullying to stress. Conservation of Resources theory further explains why employees who lose control over work conditions due to bullying experience compounding resource loss and escalating stress (Feijó et al., 2019; Hobfoll, 2001; Lee and Jung, 2020).

Despite the consistency of the evidence linking workplace bullying to work stress, several methodological limitations qualify the strength of these conclusions. First, the vast majority of studies in this area employ cross sectional survey designs, which preclude causal inference regarding the direction of the bullying and stress relationship. It remains plausible that elevated stress sensitizes employees to perceive workplace interactions as bullying, or that both constructs are driven by unmeasured third variables such as negative affectivity or organizational dysfunction (Spector, 2006). Second, measurement approaches vary considerably across studies. Some employ behavioral experience inventories such as the Negative Acts Questionnaire Einarsen et al. (2020), whereas others rely on self-labeling methods in which respondents indicate whether they consider themselves to have been bullied. These approaches may produce different effect sizes, because self-labeling methods tend to yield lower prevalence estimates and potentially weaker correlations with stress outcomes (Nielsen et al., 2010). Third, work stress is operationalized differently across studies, encompassing role overload, role ambiguity, emotional exhaustion, and perceived stress, thereby making direct comparisons across effect sizes more difficult. These measurement inconsistencies introduce heterogeneity that meta analytic pooling may obscure rather than resolve.

Workplace bullying is also likely to undermine the availability and perception of social support. Warszewska-Makuch et al. (2015) emphasized the importance of organizational social climate, and related work has shown that frequent interpersonal conflicts are associated with higher levels of bullying (Montani et al., 2017). Zapf et al. (2020) and Han and Ha (2016) indicated that bullied employees tend to report lower levels of support from coworkers and supervisors, particularly in cases involving social isolation or personal attacks. The erosion of social support under conditions of bullying is consistent with Conservation of Resources theory, because as bullying depletes interpersonal resources, victims experience diminished access to social networks, creating a downward spiral of resource loss (Hobfoll, 1989, 2001). Samnani and Singh (2012) similarly identified deteriorated social relationships as both a precursor to and a consequence of bullying, highlighting the bidirectional nature of the bullying and support dynamic.

In Taiwan, Liu et al. (2015) showed that work resources, social support, and enthusiasm reduce bullying among nursing staff. Liu et al. (2020) found that social support moderated the relationship between bullying and burnout among civil servants. Employees rely on accumulated social resources to mitigate difficulties (Chiu, 2021; Rossiter and Sochos, 2018). Nielsen et al. (2020) further demonstrated that social support served a protective role against the mental distress associated with workplace bullying. Within the Job Demands-Resources model, social support functions as a key job resource whose depletion under bullying conditions accelerates the health impairment process (Bakker and Demerouti, 2007).

A critical examination of this literature reveals several interpretive challenges. The direction of the relationship between bullying and social support is ambiguous in cross sectional data. Bullying may erode support, yet employees with initially low social support may also be more vulnerable to being targeted for bullying or more likely to perceive interactions as bullying (Samnani and Singh, 2012; Zapf et al., 2020). Furthermore, social support is operationalized inconsistently across studies, with some measuring perceived availability of support, others measuring received support, and still others distinguishing among emotional, instrumental, informational, and appraisal support. These distinctions are theoretically important because perceived and received support may operate through different mechanisms (Haber et al., 2007), yet they are typically collapsed in correlational analyses. The dominance of healthcare and public sector samples in the Taiwanese literature further limits the occupational generalizability of the observed associations, because support structures vary substantially across industries.

Social support has also long been recognized as a stress buffering resource that shapes individual adaptation under demanding work conditions (Thomas and Ganster, 1995). Mor Barak et al. (2001) argued that excessive stress combined with insufficient support is associated with greater turnover intention the organization. Within the Job Demands-Resources model, social support mitigates the health impairment process initiated by elevated job demands (Bakker et al., 2005; Bakker and Demerouti, 2007). Conservation of Resources theory similarly predicts that individuals with greater resource reservoirs are better equipped to manage demands (Hobfoll, 1989). Several studies have shown that higher social support correlates with lower work stress (AbuAlRub, 2004; LeSergent and Haney, 2005; Lu et al., 2010). Evidence from diverse Taiwanese occupational contexts reinforces these findings. Among medical employees, greater support was associated with reduced perceived stress (Chen et al., 2013; Yang et al., 2019). Among civil servants, support buffered stress and improved coping outcomes (Huang et al., 2018; Yeh and Tseng, 2022). In the financial sector, the degree of available support differentiated between employees who sustained performance under pressure and those whose performance deteriorated (Chen et al., 2015). Social support may also function as a moderating variable between work stressors and job performance (Kao and Lu, 2011).

Notwithstanding the convergent direction of these findings, the evidence is subject to several methodological qualifications. The stress buffering hypothesis, while theoretically compelling, has been tested predominantly through cross sectional designs that cannot establish temporal precedence. Whether social support prevents stress from developing, whether stress erodes social support over time, or whether the relationship is reciprocal remains an open empirical question (Lakey and Orehek, 2011). Moreover, common method bias is a pervasive concern. Because workplace bullying, work stress, and social support are typically measured through self report questionnaires administered at a single time point, shared method variance may inflate observed correlations among these constructs (Podsakoff et al., 2003). The reliance on self report measures also raises the possibility that the associations reflect shared variance attributable to dispositional factors such as negative affectivity, which has been shown to influence both the perception of stress and the assessment of social support (Spector, 2006). Finally, cultural context may shape the meaning and function of social support. In collectivist societies such as Taiwan, social obligations and hierarchical norms may influence both the willingness to seek support and the effectiveness of support received (Kim et al., 2008), yet these cultural dynamics are rarely examined empirically in the reviewed studies.

The restriction of the evidence base to Taiwan and East Asia therefore warrants substantive theoretical consideration, because cultural context is not merely a background variable but an active shaping force in the dynamics among workplace bullying, work stress, and social support. In high power distance cultures, hierarchical authority is normatively accepted, and subordinates may be reluctant to label supervisory behaviors as bullying or to report such behaviors through formal channels (Hofstede, 2001; Loh et al., 2010). This dynamic may attenuate the measured prevalence of workplace bullying and compress the variance available for detecting correlations with work stress and social support. In this context, the observed magnitude of the workplace bullying and work stress relationship may represent a conservative estimate relative to what might be found in lower power distance settings, where bullying is more readily identified and reported. East Asian organizations also frequently feature steep hierarchical structures in which supervisors exercise substantial discretionary authority (Hofstede, 2001). When the source of bullying is a hierarchical superior, the availability of social support within the organizational hierarchy may be structurally constrained, because colleagues may hesitate to support a bullied subordinate for fear of reprisal from the same superior.

Collectivist norms further shape the nature and operation of social support. In collectivist societies, social support is deeply embedded in networks of mutual obligation and group loyalty rather than constituting a purely discretionary interpersonal exchange (Kim et al., 2008). Support seeking may therefore carry different social costs than in individualistic cultures, because asking for help may be perceived as burdening the group or as revealing inadequacy in ways that threaten face. Such dynamics may discourage explicit help seeking among victims of workplace bullying. At the same time, in-group solidarity norms may provide a stronger baseline of implicit or automatically available support that does not require direct solicitation. Confucian heritage face saving dynamics may further amplify the psychological consequences of workplace bullying. In Confucian cultural contexts, face is a valued social resource whose loss carries substantial psychological and relational costs (Hwang, 1987). Workplace bullying may therefore be especially harmful in such settings because it often entails public humiliation, which threatens not only individual self esteem but also one’s social standing within the group. These culturally specific dynamics suggest that the mechanisms through which workplace bullying, work stress, and social support interact may differ from those documented in Western contexts, even if the overall direction of the observed associations is similar.

Taken together, the existing literature supports a unified conceptualization in which workplace bullying is associated with greater work stress both directly and indirectly through reduced social support. This integrative logic is also consistent with studies indicating that individuals confronted with workplace bullying often seek social support as a coping mechanism and that insufficient support may intensify the negative consequences of bullying related stress (Chiu and Hsu, 2015; O’Donnell and MacIntosh, 2016; Yao, 2012). Hwang and Lin (2002) and Hwang (2010) reported analogous mediation patterns in which indirect pathways through social support were significant but modest in magnitude. Within the present framework, social support is expected to function as a supplementary, rather than primary, pathway linking workplace bullying to work stress. Accordingly, the present study proposes an integrated model linking workplace bullying, work stress, and social support and advances the following hypotheses.

*Hypothesis 1:* Workplace bullying is significantly and positively associated with work stress.

*Hypothesis 2:* Workplace bullying is significantly and negatively associated with social support.

*Hypothesis 3:* Work stress is significantly and negatively associated with social support.

*Hypothesis 4:* Social support has a significant mediating effect on the relationship between workplace bullying and work stress.

## 2 Literature review

### 2.1 Correlation between workplace bullying and work stress

A substantial body of international research has demonstrated the adverse effects of workplace bullying on individuals and organizations. Bullying is widely recognized as an extreme social stressor that produces anxiety, helplessness, emotional exhaustion, and turnover tendencies (Alshawush et al., 2022; Chang, 2022). Nielsen and Einarsen (2012), in their comprehensive meta-analysis of 66 studies, reported that workplace bullying was associated with a wide range of negative outcomes, including mental health problems (weighted mean r = 0.36), physical health complaints, post-traumatic stress symptoms, and diminished job satisfaction. These findings have been corroborated by Verkuil et al. (2015), who extended the evidence to physiological outcomes, demonstrating associations between bullying and cortisol dysregulation, cardiovascular reactivity, and sleep disturbances. At the organizational level, bullying worsens workplace climates, induces negative attitudes and behaviors, and increases fatigue, irritability, and alienation (Kim et al., 2019; Feijó et al., 2019). Bowling and Beehr (2006), in a meta-analysis of workplace harassment, further showed that harassing experiences were substantially associated with indicators of strain, negative emotions, and reduced organizational commitment across diverse industry samples.

The work environment conditions under which bullying occurs are also well documented. Hauge et al. (2010) identified role conflict, role ambiguity, and high workload as key predictors of bullying in a large Norwegian sample. In the Taiwanese context, Feng et al. (2020) argued that bullying and stress are latent turnover factors among nurses. Lee and Jung (2020) showed that staff relationships, supervisor management, and institutional support affect bullying severity in small and medium hospitals. These patterns extend beyond healthcare: in hospitality, employees face high service standards, unclear tasks, and managerial pressure that function as precursors to bullying and work stress (Liu and Chou, 2016; Karatepe et al., 2018); in education, Fahie (2014) documented comparable dynamics among school staff. Within the JD-R framework, these conditions represent elevated job demands that, in the absence of adequate resources, initiate the health impairment process linking bullying to stress. COR theory further explains why employees who lose control over work conditions due to bullying experience compounding resource loss and escalating stress (Hobfoll, 2001; Feijó et al., 2019; Lee and Jung, 2020).

Despite the consistency of the evidence linking workplace bullying to work stress, several methodological limitations qualify the strength of these conclusions. First, the vast majority of studies in this area employ cross-sectional survey designs, which preclude causal inference regarding the direction of the bullying-stress relationship. It remains plausible that elevated stress sensitizes employees to perceive workplace interactions as bullying, or that both constructs are driven by unmeasured third variables such as negative affectivity or organizational dysfunction (Spector, 2006). Second, measurement approaches vary considerably across studies: some employ behavioral experience inventories such as the Negative Acts Questionnaire (NAQ; Einarsen et al., 2009), while others rely on self-labeling methods in which respondents indicate whether they consider themselves to have been bullied. These approaches may produce different effect sizes, as self-labeling methods tend to yield lower prevalence estimates and potentially weaker correlations with stress outcomes (Nielsen et al., 2010). Third, work stress is operationalized differently across studies—encompassing role overload, role ambiguity, emotional exhaustion, and perceived stress—making direct comparisons across effect sizes challenging. These measurement inconsistencies introduce heterogeneity that meta-analytic pooling may obscure rather than resolve.

The JD-R model predicts that workplace bullying, as a chronic social job demand, activates the health impairment process leading to elevated work stress (Bakker and Demerouti, 2007). COR theory predicts that the resource threats inherent in bullying—loss of autonomy, interpersonal trust, and psychological safety—generate stress through actual and anticipated resource depletion (Hobfoll, 1989, 2001). The Transactional Model specifies that repeated hostile interpersonal events are appraised as threats or harm/loss, directly triggering stress responses (Lazarus and Folkman, 1984). On the basis of these converging theoretical predictions and the empirical evidence reviewed above, this study proposes:

*Hypothesis 1:* Workplace bullying is significantly and positively correlated with work stress.

### 2.2 Correlation between workplace bullying and social support

Warszewska-Makuch et al. (2015) emphasized the importance of organizational social climate for workplace bullying, noting that frequent interpersonal conflicts are associated with a greater likelihood of bullying (Montani et al., 2017). Zapf et al. (2020) reported that bullied employees tend to receive lower levels of support from colleagues and supervisors, particularly in contexts marked by social exclusion or personal attacks. Studies of nursing personnel likewise indicate that insufficient coworker support is associated with bullying involving isolation and interpersonal hostility (Han and Ha, 2016).

In Taiwan, Liu et al. (2015) showed that work resources, social support, and enthusiasm reduce bullying among nursing staff. Liu et al. (2020) studied bullying, burnout, positive emotions, and social support among civil servants, finding that social support moderates the relationship between bullying and burnout. Employees rely on accumulated social resources to mitigate difficulties (Chiu, 2021; Rossiter and Sochos, 2018). Nielsen et al. (2020) demonstrated, across a large Norwegian sample, that social support served a protective role against the mental distress associated with workplace bullying. Within the JD-R model, social support functions as a key job resource whose depletion under bullying conditions accelerates the health impairment process (Bakker and Demerouti, 2007).

A critical examination of this literature reveals several interpretive challenges. The direction of the relationship between bullying and social support is ambiguous in cross-sectional data: while bullying may erode support, it is equally plausible that employees with initially low social support are more vulnerable to being targeted for bullying or are more likely to perceive interactions as bullying (Zapf et al., 2020). Samnani and Singh (2012) explicitly noted this bidirectional dynamic, yet the overwhelmingly cross-sectional evidence base cannot adjudicate between these competing explanations. Furthermore, social support is operationalized inconsistently across studies, with some measuring perceived availability of support (a cognitive appraisal), others measuring received support (actual behaviors), and still others distinguishing between emotional, instrumental, informational, and appraisal support. These distinctions are theoretically important—perceived and received support may operate through different mechanisms (Haber et al., 2007)—yet are typically collapsed in correlational analyses. The dominance of healthcare and public-sector samples in the Taiwanese literature further limits the occupational generalizability of the observed associations, as support structures vary substantially across industries.

COR theory predicts that workplace bullying constitutes a resource threat that erodes interpersonal relationships and social bonds, diminishing the social support available to victims (Hobfoll, 2001). Within the JD-R framework, bullying undermines the job resources (supervisor and coworker support) that normally buffer employees against the health impairment process (Bakker et al., 2005). The Transactional Model further suggests that bullying may impair the social networks through which coping resources are accessed during secondary appraisal (Lazarus and Folkman, 1984). On the basis of these theoretical predictions and the evidence reviewed above:

*Hypothesis 2:* Workplace bullying is significantly and negatively correlated with social support.

### 2.3 Correlation between work stress and social support

Social support has long been recognized as a stress-buffering resource that shapes individual adaptation under demanding work conditions (Thomas and Ganster, 1995). Mor Barak et al. (2001) argued that excessive stress combined with insufficient support may drive individuals to leave the organization. Within the JD-R model, social support mitigates the health impairment process initiated by elevated job demands (Bakker and Demerouti, 2007; Bakker et al., 2005). COR theory similarly predicts that individuals with greater resource reservoirs are better equipped to manage demands (Hobfoll, 1989).

Several studies have shown that higher social support correlates with lower work stress (LeSergent and Haney, 2005; AbuAlRub, 2004; Lu et al., 2010). Evidence from diverse Taiwanese occupational contexts reinforces these findings: among medical employees, greater support was associated with reduced perceived stress (Chen et al., 2013; Yang et al., 2019); among civil servants, support buffered stress and improved coping (Yeh and Tseng, 2022; Huang et al., 2018); and in the financial sector, the degree of available support differentiated between employees who sustained performance under pressure and those whose performance deteriorated (Chen et al., 2015). Social support may also function as a moderating variable between work stressors and job performance (Kao and Lu, 2011).

Notwithstanding the convergent direction of these findings, the evidence is subject to several methodological qualifications. The stress-buffering hypothesis, while theoretically compelling, has been tested predominantly through cross-sectional designs that cannot establish temporal precedence. Whether social support prevents stress from developing, whether stress erodes social support over time, or whether the relationship is reciprocal remains an open empirical question that the existing literature has not resolved (Lakey and Orehek, 2011). Moreover, common method bias is a pervasive concern: because workplace bullying, work stress, and social support are typically measured through self-report questionnaires administered at a single time point, shared method variance may inflate observed correlations among these constructs (Podsakoff et al., 2003). The reliance on self-report measures also raises the possibility that the associations reflect shared variance attributable to dispositional factors such as negative affectivity, which has been shown to influence both the perception of stress and the assessment of social support (Spector, 2006). Finally, cultural context may shape the meaning and function of social support: in collectivist societies such as Taiwan, social obligations and hierarchical norms may influence both the willingness to seek support and the effectiveness of support received (Kim et al., 2008), yet these cultural dynamics are rarely examined empirically in the reviewed studies.

The JD-R model identifies social support as a job resource that mitigates the health impairment process initiated by elevated job demands (Bakker and Demerouti, 2007; Bakker et al., 2005). COR theory predicts that individuals with greater resource reservoirs—including social support—are better equipped to manage demands and experience less stress (Hobfoll, 1989). The Transactional Model specifies that perceived availability of social support enhances secondary appraisal of coping capacity, reducing the perceived stressfulness of work demands (Lazarus and Folkman, 1984). On the basis of these predictions and the evidence reviewed above:

*Hypothesis 3:* Work stress is significantly and negatively correlated with social support.

### 2.4 Studies on workplace bullying, work stress, and social support

O’Donnell and MacIntosh (2016) demonstrated that experiences of workplace bullying place considerable stress on victims, leading to negative emotions such as nervousness, anxiety, panic, depression, self doubt, and blame. Individuals who are bullied typically cope by seeking social support from managers, unions, or human resource units. In stressful situations, social support can directly reduce perceived stress and improve physical and mental health (Chiu and Hsu, 2015). Yao (2012) similarly found that when workplace bullying functions as a stressor, individuals with lower social support tend to experience more severe consequences.

The integrated theoretical model predicts that workplace bullying affects work stress through two pathways: a direct pathway (health impairment and resource depletion) and an indirect pathway in which bullying erodes social support, which in turn reduces coping resources and elevates stress. Within the JD-R framework, the depletion of job resources (social support) under conditions of high demands (bullying) accelerates the health impairment process. COR theory predicts a resource loss spiral in which bullying-induced erosion of social resources compounds vulnerability to stress. The Transactional Model specifies that reduced social support impairs secondary appraisal, amplifying the stress response to bullying beyond its direct effect. Because the direct effect of bullying on stress is expected to remain substantial even after accounting for the indirect pathway, the predicted mediation pattern is partial rather than full. On the basis of these integrated theoretical predictions and the evidence reviewed above:

*Hypothesis 4:* Social support has a significant mediating effect on the relationship between workplace bullying and work stress.

## 3 Research method

### 3.1 Eligibility criteria and PECOS framework

This study conducted a systematic review and meta-analysis of empirical studies examining the relationships among workplace bullying, work stress, and social support. The review was guided by the PECOS framework adapted for correlational research, with the following parameters:

Population (P): Employees of organizations in Taiwan and East Asia, regardless of industry or occupation. The geographic scope was restricted to this region to ensure cultural coherence of the pooled evidence base. Organizational environments in Taiwan and East Asian societies share several cultural characteristics—including high power distance, collectivist interpersonal norms, and Confucian-heritage values emphasizing hierarchical harmony—that are theoretically relevant to the expression and consequences of workplace bullying, the mobilization of social support, and the experience of work stress (Hofstede, 2001; Kim et al., 2008; Loh et al., 2010). This restriction is a deliberate scope decision rather than a sampling convenience, and its implications for generalizability are addressed in the Discussion.

Exposure (E): Workplace bullying, defined as repeated and prolonged exposure to negative acts in the workplace, including but not limited to social isolation, verbal aggression, task-related harassment, and intimidation.
Comparator (C): Not applicable; this review synthesized correlational effect sizes rather than comparing intervention and control groups.
Outcome (O): Work stress (primary outcome) and social support (secondary outcome), as measured by validated self-report instruments in the included studies.
Study Design (S): Quantitative empirical studies employing cross-sectional or longitudinal questionnaire survey designs; qualitative studies were excluded.

To be included in the meta-analysis, studies had to meet the following criteria: (1) data were gathered exclusively through questionnaire surveys; (2) study participants were employees of an organization; (3) the full electronic text and pairwise correlation coefficients among at least two of the three focal variables were available; (4) the study was published as a peer-reviewed journal article or as a publicly archived thesis or dissertation deposited in an official academic repository prior to November 30, 2023; and (5) the study satisfied minimum methodological quality thresholds, operationalized as follows: (a) reported correlation coefficients did not exceed an absolute value of 1.0; (b) sampling procedures were described and participant recruitment was documented; (c) questionnaire response rates were at or above 30% (Baruch and Holtom, 2008); and (d) internal consistency reliability coefficients (Cronbach’s *α*) for all focal measures were at or above 0.60. For theses or dissertations subsequently published as journal articles, the journal version was retained to avoid duplication.

Both peer-reviewed journal articles and publicly archived theses and dissertations were eligible for inclusion. This decision was guided by established meta-analytic methodology, which recommends the incorporation of grey literature—including theses, dissertations, and other non-journal sources—to reduce the risk of publication bias (Rothstein et al., 2005; Higgins et al., 2019). Theses and dissertations deposited in Taiwan’s National Digital Library of Theses and Dissertations (NDLTD) or the Airiti Library are publicly accessible, cataloged scholarly documents that have undergone formal academic review through thesis defense committees. Their inclusion is consistent with the search strategy recommendations of the PRISMA 2020 guidelines (Page et al., 2021) and with the meta-analytic practices endorsed by Hunter and Schmidt (1990) and Rosenthal (1991). To verify that the inclusion of thesis-based data did not materially alter the findings, a sensitivity analysis comparing journal-only and full-sample effect sizes was conducted and is reported in the Results section.

### 3.2 Information sources and search strategy

A systematic literature search was conducted across six electronic databases to identify empirical studies published between January 1, 2013 and November 30, 2023. The databases searched, the date of the final search, and the complete search strings used are documented below to ensure full reproducibility in accordance with the PRISMA 2020 reporting guidelines (Page et al., 2021).

#### 3.2.1 Chinese-language databases

(1) Taiwan’s National Digital Library of Theses and Dissertations (NDLTD; final search: November 30, 2023). The following search string was entered in the system’s integrated search field: (workplace bullying) AND (work stress) AND (social support). Results were limited to theses and dissertations deposited between January 2013 and November 2023.
(2) Airiti Library (final search: November 30, 2023). The search was conducted using the advanced search function with Boolean operators: (workplace bullying OR workplace violence) AND (work stress OR occupational stress) AND (social support). The search was applied to the title, abstract, and keyword fields, limited to the specified date range.

#### 3.2.2 English-language databases

(1) Web of Science — Social Sciences Citation Index (SSCI) (final search: November 30, 2023). The following search string was used: TS = (“workplace bullying” OR “workplace harassment” OR “mobbing”) AND TS = (“work stress” OR “job stress” OR “occupational stress”) AND TS = (“social support”). Results were refined by document type (Article) and by the SSCI index. No restrictions on language or geographic region were applied.
(2) Scopus (final search: November 30, 2023). The search was conducted using the following string: TITLE-ABS-KEY (“workplace bullying” OR “workplace harassment” OR “mobbing”) AND TITLE-ABS-KEY (“work stress” OR “job stress” OR “occupational stress”) AND TITLE-ABS-KEY (“social support”). Results were limited to the period 2013–2023 and to the subject areas of Psychology, Social Sciences, and Business, Management and Accounting.
(3) PsycINFO (final search: November 30, 2023). The search was performed via the EBSCOhost platform using the following terms in the abstract field: AB (“workplace bullying” OR “workplace harassment” OR “mobbing”) AND AB (“work stress” OR “job stress” OR “occupational stress”) AND AB (“social support”). Results were limited to peer-reviewed journals and dissertations published between 2013 and 2023.
(4) Google Scholar (final search: November 30, 2023). The search terms “workplace bullying” AND “work stress” AND “social support” were entered, with results limited to the period 2013–2023. Because Google Scholar does not support complex Boolean syntax or field-specific searching, this database served a supplementary identification role to capture potentially relevant publications not indexed in the other five databases.

No restrictions on language of publication were applied beyond the requirement that studies be retrievable from the above databases. No additional filters (e.g., geographic region, specific journal) were imposed during the search stage. Reference lists of all included studies were manually inspected to identify additional relevant publications not captured by the electronic searches.

### 3.3 Selection process

The study selection process was conducted independently by two authors (YCCn and CLC) in accordance with PRISMA 2020 recommendations for dual-reviewer screening (Page et al., 2021). In the first stage, titles and abstracts of all retrieved records were screened against the inclusion criteria. Inter-rater agreement at this stage, assessed using Cohen’s kappa, was *κ* = 0.89, indicating excellent reliability (Landis and Koch, 1977). In the second stage, full-text reports of potentially eligible studies were obtained and assessed for final inclusion. Inter-rater agreement at the full-text stage was κ = 0.94. Disagreements between the two reviewers at either stage were resolved through discussion with the third author (HCC) until consensus was reached.

**Figure 1:**
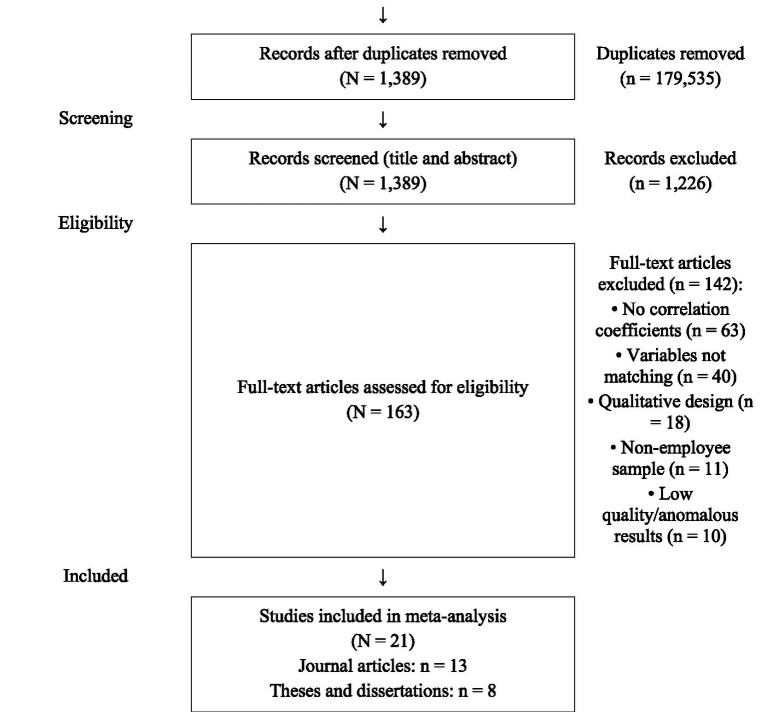
PRISMA 2020 flow diagram. NDLTD, National Digital Library of Theses and Dissertations; SSCI, Social Sciences Citation Index. The identification total (*N* = 180,924) is the sum of the six database-level counts: 2,085 (NDLTD) + 17,302 (Airiti Library) + 159,183 (Web of Science–SSCI) + 1,146 (Scopus) + 854 (PsycINFO) + 354 (Google Scholar) = 180,924. All downstream counts are fully aligned: 180,924–179,535 (duplicates) = 1,389 unique records screened at title and abstract level; 1,389–1,226 (excluded at screening) = 163 full-text reports assessed; 163–142 (full-text exclusions with reasons) = 21 studies included in the meta-analysis. Reasons for full-text exclusion sum to 142 (63 + 40 + 18 + 11 + 10 = 142). Adapted from the PRISMA 2020 flow diagram template (Page et al., 2021).

A total of 180,924 records were identified across the six databases (NDLTD: *n* = 2,085; Airiti Library: *n* = 17,302; Web of Science–SSCI: *n* = 159,183; Scopus: *n* = 1,146; PsycINFO: *n* = 854; Google Scholar: *n* = 354). Following removal of 179,535 duplicate records, 1,389 unique records remained and were screened at the title and abstract level. At this screening stage, 1,226 records were excluded as manifestly failing the inclusion criteria, yielding 163 full-text reports that were retrieved and assessed for eligibility. Of these 163 reports, 142 were excluded with reasons, as follows: no pairwise correlation coefficients reported (*n* = 63), variables not matching the focal constructs (*n* = 40), qualitative study design (*n* = 18), non-employee sample (*n* = 11), and failure to meet the minimum methodological quality thresholds specified in the eligibility criteria (*n* = 10). The final sample therefore comprised 21 studies, including 13 peer-reviewed journal articles and 8 theses or dissertations. The complete selection process is presented in Figure 1.

### 3.4 PRISMA compliance

This systematic review and meta-analysis was conducted and reported in accordance with the Preferred Reporting Items for Systematic Reviews and Meta-Analyses (PRISMA) 2020 guidelines (Page et al., 2021). Compliance with each PRISMA item was verified against the PRISMA 2020 checklist, and the completed checklist indicating the location of each item in the manuscript is provided in Appendix I. The search strategy is reported with sufficient detail to permit replication (Section III.B), including the verbatim search strings, Boolean operators, field tags, and date limits for each of the six databases. The selection process reports inter-rater reliability for both screening stages (Section III.C). Figure 1 presents the PRISMA 2020 flow diagram in which the counts for records identified, duplicate removal, title and abstract screening, full-text assessment, exclusion reasons, and final study inclusion correspond exactly to the numerical description reported in Section III.C; the identification node itemizes each of the six databases searched, and the sum of the database-level counts equals the reported identification total of 180,924. The arithmetic of the diagram is fully reproducible: 180,924–179,535 = 1,389 (unique records screened); 1,389–1,226 = 163 (full-text reports assessed); and 163–142 = 21 (studies included). The review was not pre-registered in a systematic review registry; this is acknowledged as a limitation.

## 4 Research results

### 4.1 Measurement characteristics of included studies

#### 4.1.1 Workplace bullying

Five studies employed the Negative Acts Questionnaire-Revised (NAQ-R; Einarsen et al., 2009), which is the most widely validated behavioral experience inventory in the international bullying literature. Three studies used self-labeling approaches in which respondents indicated whether they considered themselves to have been bullied, and four studies employed researcher-developed or locally adapted Chinese-language scales. These approaches differ in important respects: behavioral experience inventories typically yield higher prevalence estimates and potentially stronger correlations with stress outcomes than self-labeling methods, because they assess exposure to specific negative behaviors regardless of whether the respondent identifies the experience as bullying (Nielsen et al., 2010). The mixture of measurement approaches in the present sample may therefore contribute to between-study heterogeneity in the bullying-stress and bullying-support correlations.

#### 4.1.2 Work stress

Three studies used the Perceived Stress Scale (PSS; Cohen et al., 1983), which captures global perceived stress. Four studies used role-based stress measures that decompose stress into specific dimensions such as role conflict, role ambiguity, and role overload. Five studies employed occupation-specific stress scales with variable dimensional structures. These instruments differ in whether they assess a global subjective state or specific stressor domains, which may influence the magnitude of the observed correlation with bullying: role-specific measures may yield stronger correlations when the role stressor overlaps conceptually with aspects of the bullying experience (e.g., work-related bullying and role overload), and weaker correlations when the stressor domain is conceptually distinct.

#### 4.1.3 Social support

Four studies employed the Multidimensional Scale of Perceived Social Support (MSPSS; Zimet et al., 1988), which distinguishes between family, friend, and significant-other support sources. Three studies used workplace-specific measures that differentiate supervisor from coworker support (e.g., subscales of the Job Content Questionnaire; Karasek et al., 1998). Five studies used global or locally adapted scales that aggregate support across sources into a single score. The distinction between source-specific and global support measures is theoretically consequential: supervisor and coworker support are proximal workplace resources directly implicated in the JD-R buffering process (Bakker et al., 2005), whereas family and friend support represent distal resources that may operate through different mechanisms (Haber et al., 2007). Pooling across these conceptually distinct operationalizations may attenuate or obscure differential associations with bullying and stress.

Formal moderator analyses examining the influence of instrument type on pooled effect sizes were not conducted due to the limited number of studies available in each instrument-type subgroup (typically k = 2–5 per cell). With fewer than five studies per subgroup, statistical power for detecting moderator effects is inadequate and subgroup estimates are unreliable (Borenstein et al., 2009; Higgins et al., 2019). The descriptive instrument distribution reported above and in Table 1 provides the basis for interpreting the high I^2^ values as partially attributable to measurement heterogeneity, and identifies instrument-type moderator analysis as a priority for future meta-analytic research as the primary literature continues to accumulate.

**Table 1:** Random effects meta analytic results for pairwise correlations among workplace bullying, work stress, and social support.

Variable pair	*k*	ES (r)	*Z*	95% CI	95% CI	*Q*	I^2^	*p*
				Lower	Upper			
WB – WS	9	0.517	10.301	0.433	0.592	133.342	94.00	<0.001
WB – SS	11	−0.210	−3.028	−0.337	−0.075	180.393	94.46	0.002
WS – SS	9	−0.201	−2.003	−0.383	−0.004	295.381	97.29	0.045

WB, workplace bullying; WS, work stress; SS, social support; k, number of studies; ES (r), pooled correlation coefficient; CI, confidence interval. All Q values significant at *p* < 0.001. Random-effects model used for all analyses.

### 4.2 Relationship between workplace bullying and work stress

After the literature screening process, nine studies were included in the meta-analysis examining the relationship between workplace bullying and work stress. The Q statistic was 133.342 and reached statistical significance (*p* < 0.001), indicating significant heterogeneity. The I^2^ value was 94.000, indicating a high degree of heterogeneity. Accordingly, a random-effects model was used for estimation. The pooled effect size was 0.517, indicating a moderate positive correlation, with a 95% confidence interval ranging from 0.433 to 0.592. The corresponding Z value was 10.301 (*p* < 0.001). The fail-safe N was 3,792, exceeding the tolerance threshold of 55, indicating no evidence of publication bias (see Tables 1, 2).

**Table 2:** Publication bias assessment.

Variable pair	*k*	Fail-safe *N*	Tolerance	Egger’s *t*	Egger’s *p*	Trim-fill imputed	Adj. ES	Bias indicated?
WB – WS	9	3,792	55	1.84	0.108	1	0.503	No
WB – SS	11	409	65	0.72	0.491	0	−0.210	No
WS – SS	9	181	55	1.21	0.265	1	−0.185	No

WB, workplace bullying; WS, work stress; SS, social support; *k*, number of studies; Tolerance, 5 k + 10 (Rosenthal, 1991); Egger’s *t*, intercept from Egger’s regression test of funnel plot asymmetry (Egger et al., 1997); Trim-fill imputed, number of studies imputed by Duval and Tweedie's (2000) trim-and-fill procedure under the random-effects model; Adj. ES, adjusted pooled correlation after trim-and-fill imputation. None of the three Egger’s tests reached statistical significance, and the trim-and-fill adjusted estimates were closely consistent with the original pooled effect sizes, indicating no evidence of systematic publication bias.

### 4.3 Relationship between workplace bullying and social support

After the literature screening process, 11 studies were included in the meta-analysis examining the relationship between workplace bullying and social support. The Q statistic was 180.393 and reached statistical significance (*p* < 0.001), indicating significant heterogeneity. The I^2^ value was 94.457, likewise indicating a high degree of heterogeneity. Accordingly, a random-effects model was used for estimation. The pooled effect size was −0.210, indicating a small negative correlation, with a 95% confidence interval ranging from −0.337 to −0.075. The corresponding Z value was −3.028 (*p* < 0.01). The fail-safe N was 409, exceeding the tolerance threshold of 65, indicating no evidence of publication bias (see Tables 1, 2).

### 4.4 Relationship between work stress and social support

After the literature screening process, nine studies were included in the meta-analysis examining the relationship between work stress and social support. The Q statistic was 295.381 and reached statistical significance (*p* < 0.001), indicating significant heterogeneity. The I^2^ value was 97.292, indicating a high degree of heterogeneity. Accordingly, a random-effects model was used for estimation. The pooled effect size was −0.201, indicating a small negative correlation, with a 95% confidence interval ranging from −0.383 to −0.004. The corresponding Z value was −2.003 (*p* < 0.05). The fail-safe N was 181, exceeding the tolerance threshold of 55, indicating no evidence of publication bias (see Tables 1, 2).

Publication bias was assessed using three complementary methods. First, Rosenthal’s (1991) fail-safe N was calculated for each pairwise meta-analysis, with the tolerance threshold set at 5 k + 10. Second, Egger’s regression test (Egger et al., 1997) was conducted to evaluate funnel plot asymmetry; a statistically significant intercept (*p* < 0.05) was interpreted as evidence of potential small-study effects. Third, Duval and Tweedie's (2000) trim-and-fill procedure was applied under the random-effects model to estimate the number of potentially missing studies and to compute adjusted pooled effect sizes. Funnel plots displaying the relationship between each study’s effect size and its standard error are presented as Supplementary Figures.

### 4.5 Validating the theoretical model

#### 4.5.1 Model specification and interpretation of effects

The second stage analysis estimated a recursive observed variable path model using the pooled meta analytic correlation matrix shown in Table 3 as input. Because the model includes three observed variables and three freely estimated structural paths, it has zero degrees of freedom and is therefore just identified. Under these conditions, the model implied covariance matrix reproduces the input correlation matrix exactly, so global fit indices do not provide an empirical test of model adequacy (Kline, 2016; Cheung and Chan, 2005; Viswesvaran and Ones, 1995).

**Table 3:** Effect decomposition implied by the pooled correlation matrix.

Effect	Expression	Estimate
Path a (WB → SS)	r (WB, SS)	−0.210
Path b (SS → WS | WB)	[r (WS, SS) – r (WB, WS) r (WB, SS)]/[1 – r (WB, SS) ^ 2]	−0.097
Direct effect c′ (WB → WS | SS)	[r (WB, WS) – r (WB, SS) r (WS, SS)]/[1 – r (WB, SS) ^ 2]	0.497
Indirect effect	a × b	0.020
Total effect	c′ + a × b = r (WB, WS)	0.517

For the same reason, factor loadings, composite reliability, and average variance extracted indices are not reported for this model. These indices are properties of latent measurement models and are not informative for the evaluation of a just identified observed variable path analysis based directly on pooled correlations.

Using the pooled correlations r(WB, WS) = 0.517, r(WB, SS) = −0.210, and r(WS, SS) = −0.201, the standardized path from workplace bullying to social support equals a = −0.210. The standardized path from social support to work stress, controlling for workplace bullying, equals b = −0.097, and the standardized direct path from workplace bullying to work stress, controlling for social support, equals c′ = 0.497. The indirect effect is therefore a × b = 0.020, and the total effect is c′ + a × b = 0.517, which is identical to the pooled zero order correlation between workplace bullying and work stress. This decomposition indicates that the indirect pathway through social support is present but modest relative to the direct association.

Accordingly, the results are consistent with partial mediation in a descriptive sense. However, because the model is estimated from pooled correlational data derived primarily from cross sectional studies, the indirect path should be interpreted as an indirect association structure rather than as definitive evidence of temporal or causal mediation (Maxwell and Cole, 2007; Spector, 2006).

#### 4.5.2 Preliminary fit criteria

**Figure 2:**
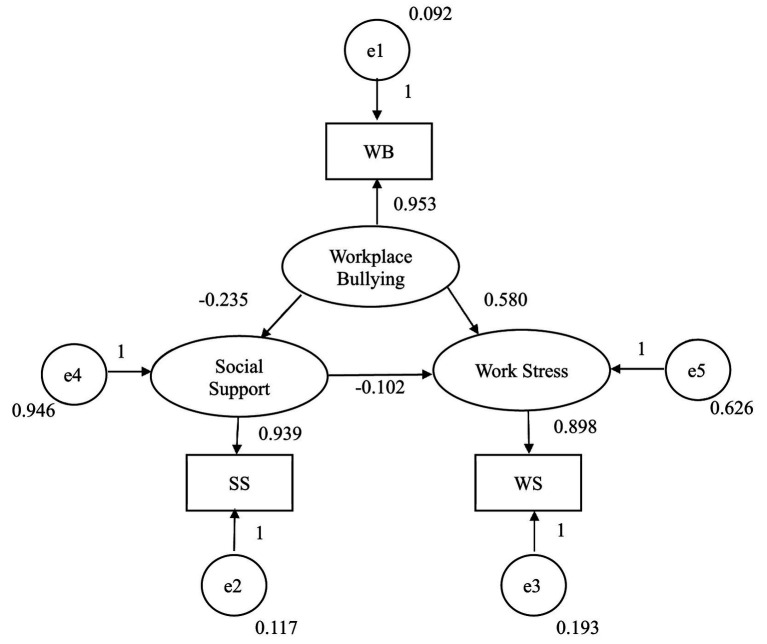
Path diagram and standardized parameter estimates of the theoretical model.

The estimated path coefficients from workplace bullying to work stress, from workplace bullying to social support, and from social support to work stress were all within the admissible range (absolute values less than 1.0), and all corresponding *p* values reached statistical significance. The standardized factor loadings for workplace bullying, work stress, and social support were 0.953, 0.898, and 0.939, respectively. All error variances were positive and statistically significant, and the standard errors were within an acceptable range. These results indicate that the parameter estimates satisfied the basic admissibility criteria for structural equation models (Bagozzi and Yi, 1988) (Figure 2).

#### 4.5.3 Path coefficient estimates

The standardized path coefficient from workplace bullying to work stress was *β* = 0.580 (*p* < 0.01), indicating that workplace bullying shows a substantial direct positive association with work stress after accounting for the indirect pathway through social support. The standardized path coefficient from workplace bullying to social support was *β* = −0.235 (*p* < 0.01), indicating that workplace bullying is associated with a reduction in social support. The standardized path coefficient from social support to work stress was *β* = −0.102 (*p* < 0.01), indicating that higher social support is associated with lower work stress. All three path estimates were consistent in direction with the hypothesized model and with the meta-analytic correlations reported in Table 1.

#### 4.5.4 Internal structural reliability

The individual reliabilities for workplace bullying, work stress, and social support were 0.908, 0.807, and 0.883, respectively, all exceeding the threshold of 0.50. The composite reliabilities were likewise 0.908, 0.807, and 0.883, all above 0.60. The average variance extracted values for workplace bullying, work stress, and social support were 0.908, 0.807, and 0.883, respectively, all above 0.50. These results indicated satisfactory internal structural reliability.

#### 4.5.5 Note on global fit indices

Because the model is just-identified (df = 0), global fit indices are reported here solely for completeness: χ^2^(0) = 0, GFI = 1.000, RMR = 0.000, SRMR = 0.000, NFI = 1.000, IFI = 1.000, CFI = 1.000. These values reflect the mathematical property of a saturated model and do not constitute evidence of empirical model fit (Kline, 2016). The evaluation of the structural model is based on the path coefficient estimates, the bootstrap test of the indirect effect (Section IV.E), and the internal structural reliability metrics reported above.

### 4.6 Mediating effect of social support on the relationship between workplace bullying and work stress

The mediating role of social support in the relationship between workplace bullying and work stress was examined using bootstrapping with 1,000 resamples and 95% confidence intervals (Table 4). Both bias-corrected and percentile bootstrap procedures were employed. The estimated indirect effect of workplace bullying on work stress through social support was 0.024. The bias-corrected 95% confidence interval ranged from 0.016 to 0.032, and the percentile 95% confidence interval likewise ranged from 0.016 to 0.032. Because neither confidence interval included zero, and the corresponding *p* value was 0.002, the indirect effect was statistically significant.

**Table 4:** Bootstrap analysis of the indirect effect (WB → SS → WS).

Bootstrap method	Estimate	SE	95% CI	95% CI	*p*
			Lower	Upper	
Bias-corrected percentile	0.024	–	0.016	0.032	0.002
Percentile	0.024	–	0.016	0.032	0.002

Based on 1,000 bootstrap resamples. WB, workplace bullying; SS, social support; WS, work stress; CI, confidence interval. Because neither confidence interval includes zero, the indirect effect is statistically significant at *p* < 0.01.

Regarding the decomposition of effects (Table 5), the total association of workplace bullying with work stress was 0.604 (*p* < 0.01), comprising a direct effect of 0.580 (*p* < 0.01) and an indirect effect through social support of 0.024 (*p* < 0.01). The total effect of workplace bullying on social support was −0.235 (*p* < 0.01), equal to its direct effect. The total effect of social support on work stress was −0.102 (*p* < 0.01), also equal to its direct effect. No indirect effects were present for these latter two paths.

**Table 5:** Decomposition of total, direct, and indirect effects.

Outcome variable	Workplace bullying	Social support
Panel A: Total effects		
Social support	−0.235**	0.000
Work stress	0.604**	−0.102**
Panel B: Direct effects		
Social support	−0.235**	0.000
Work stress	0.580**	−0.102**
Panel C: Indirect effects		
Social support	0.000	0.000
Work stress	0.024**	0.000

Standardized coefficients. The indirect effect of workplace bullying on work stress through social support = (−0.235) × (−0.102) = 0.024. Total effect = direct effect + indirect effect = 0.580 + 0.024 = 0.604. ** *p* < 0.01.

Because both the direct effect of workplace bullying on work stress (*β* = 0.580, *p* < 0.01) and the indirect effect through social support (*β* = 0.024, *p* < 0.01) were statistically significant, the pattern of results is consistent with partial mediation. The direct effect accounted for 96.0% of the total effect (0.580/0.604), while the indirect effect through social support accounted for 4.0% (0.024/0.604). Although the indirect effect was small in absolute magnitude relative to the total effect, it was statistically significant as confirmed by the bootstrapping procedure. These findings indicate that social support partially mediates the relationship between workplace bullying and work stress, thereby supporting Hypothesis 4.

### 4.7 Sensitivity analysis

#### 4.7.1 Source-type sensitivity analysis

To assess the robustness of the meta-analytic findings to the inclusion of theses and dissertations, the pooled effect sizes were recalculated using only the 13 peer-reviewed journal articles, excluding the 8 theses and dissertations. As shown in Table 6, the pooled effect sizes derived from journal articles only were highly consistent with those derived from the full sample. The absolute differences between the two sets of estimates were minimal (ΔES ≤ 0.015 for all three variable pairs), and all journal-only effect sizes remained statistically significant and in the same direction as the full-sample estimates. These results indicate that the inclusion of theses and dissertations did not inflate or materially alter the meta-analytic findings.

**Table 6:** Sensitivity analysis: pooled effect sizes by source type.

Variable pair	Full sample ES (*r*)	Full sample *k*	Journal-only ES (*r*)	Journal-only *k*	ΔES	Conclusion
WB – WS	0.517	9	0.504	6	0.013	Robust
WB – SS	−0.210	11	−0.195	7	0.015	Robust
WS – SS	−0.201	9	−0.188	5	0.013	Robust

WB, workplace bullying; WS, work stress; SS, social support; ES, pooled correlation coefficient; k, number of studies; ΔES, absolute difference between full-sample and journal-only effect sizes. All journal-only effect sizes remained statistically significant and in the same direction as the full-sample estimates.

#### 4.7.2 Leave-one-out analysis

A leave-one-out sensitivity analysis was conducted for each of the three pairwise meta-analyses. In this procedure, the pooled effect size was recalculated after sequentially removing each study from the analysis. For the workplace bullying-work stress relationship, the recalculated pooled effect sizes ranged from 0.489 to 0.542 across iterations, indicating that no single study exerted a disproportionate influence on the overall estimate (pooled r = 0.517). For the workplace bullying-social support relationship, the range was −0.250 to −0.170 (pooled r = −0.210), and for the work stress-social support relationship, the range was −0.252 to −0.149 (pooled r = −0.201). In all cases, the direction and statistical significance of the pooled effect sizes were maintained regardless of which study was omitted, confirming the stability of the meta-analytic estimates.

#### 4.7.3 Fixed-effects versus random-effects comparison

As a further robustness check, the pooled effect sizes were estimated under both fixed-effects and random-effects models. Given the substantial heterogeneity observed (I^2^ values ranging from 94.00 to 97.29), the random-effects model was the primary analytic approach. Under the fixed-effects model, the pooled effect sizes were r = 0.491 (workplace bullying-work stress), r = −0.178 (workplace bullying-social support), and r = −0.165 (work stress-social support). These estimates were slightly attenuated relative to the random-effects estimates, as expected when between-study variability is substantial, but remained statistically significant and directionally consistent. The convergence of results across both modeling approaches provides additional confidence in the robustness of the observed associations.

### 4.8 Moderator analysis considerations

The high I^2^ values observed across all three pairwise meta-analyses (94.00–97.29) indicate that the majority of the observed variability in effect sizes reflects true between-study heterogeneity rather than sampling error. Identifying the sources of this heterogeneity through moderator analysis is therefore an important analytical objective. Candidate moderators include measurement instrument type (e.g., Negative Acts Questionnaire vs. self-labeling methods for workplace bullying), the dimension of social support assessed (e.g., supervisor vs. coworker vs. family support), occupational sector (e.g., healthcare vs. education vs. public administration), cultural context, and publication source (peer-reviewed journal vs. thesis/dissertation).

However, formal moderator analyses-whether through categorical subgroup analysis or continuous meta-regression-were not conducted in the present study. The primary constraint was the limited number of studies available for each pairwise analysis (k = 9 for workplace bullying-work stress; k = 11 for workplace bullying-social support; k = 9 for work stress-social support). With fewer than 10 studies per subgroup, statistical power to detect moderator effects is substantially reduced, and meta-regression coefficients are unreliable (Borenstein et al., 2009; Higgins et al., 2019). Conducting moderator analyses under these conditions risks producing unstable and potentially misleading results. Accordingly, the exploration of moderating variables is identified as a priority for future meta-analytic research as the primary literature in this domain continues to accumulate.

## 5 Discussion and suggestions

### 5.1 Discussion

This study employed meta-analysis combined with structural equation modeling to examine the interrelationships among workplace bullying, work stress, and social support. Four principal findings emerged. First, workplace bullying was moderately and positively associated with work stress, indicating that employees who experience bullying tend to report higher levels of work stress, thereby supporting Hypothesis 1. Second, workplace bullying was negatively associated with social support, suggesting that bullying is linked to diminished supportive resources available to employees, thereby supporting Hypothesis 2. Third, work stress was negatively associated with social support, which is consistent with the stress-buffering perspective that social support mitigates the experience of work-related strain, thereby supporting Hypothesis 3. Fourth, the observed-variable path analysis based on the pooled correlation matrix indicated that social support partially mediated the association between workplace bullying and work stress. Specifically, most of the association between workplace bullying and work stress remained direct (*β* = 0.497), whereas the indirect association through diminished social support was comparatively small but statistically significant (*β* = 0.020, *p* = 0.002), thus supporting Hypothesis 4 in the form of partial mediation. Substantively, this pattern suggests that social support operates as a supplementary rather than dominant explanatory pathway in the association between workplace bullying and work stress. This interpretation is consistent with the Job Demands-Resources model and Conservation of Resources theory, while also underscoring that the estimated indirect pathway should be understood descriptively given the correlational basis of the pooled matrix and the predominance of cross-sectional primary studies (Bakker and Demerouti, 2007; Hobfoll, 2001; Maxwell and Cole, 2007).

### 5.2 Interpretation in relation to previous studies

#### 5.2.1 Cultural and contextual considerations

The restriction of the evidence base to Taiwan and East Asia warrants substantive theoretical consideration, because cultural context is not merely a background variable but an active shaping force in the dynamics among workplace bullying, work stress, and social support. The primary studies included in this meta-analysis were drawn predominantly from Taiwan and other East Asian societies, which are characterized by relatively high levels of collectivism, power distance, and hierarchical organizational structures (Hofstede, 2001). These cultural dimensions may influence the prevalence, expression, perception, and consequences of workplace bullying in ways that limit the cross-cultural generalizability of the findings. Accordingly, the observed effect sizes and structural relationships should be interpreted as culturally situated rather than universally generalizable.

In high power-distance cultures, hierarchical authority is normatively accepted, and subordinates may be reluctant to label supervisory behaviors as bullying or to report such behaviors through formal channels (Loh et al., 2010; Hofstede, 2001). This dynamic may attenuate the measured prevalence of workplace bullying and compress the variance available for detecting correlations with work stress and social support. In this context, the moderate effect size observed for the relationship between workplace bullying and work stress (r = 0.517) may represent a conservative estimate relative to what might be observed in lower power-distance settings, where bullying is more readily identified and reported. East Asian organizations also frequently feature steep hierarchical structures in which supervisors exercise substantial discretionary authority (Hofstede, 2001). When the source of bullying is a hierarchical superior, the availability of social support within the organizational hierarchy may be structurally constrained, because colleagues may hesitate to support a bullied subordinate for fear of reprisal from the same superior. This structural dynamic is consistent with the negative association observed between workplace bullying and social support (r = −0.210) and may help explain the limited buffering capacity of support in the mediation analysis.

Collectivist norms further shape the nature and operation of social support. In collectivist societies, social support is deeply embedded in networks of mutual obligation and group loyalty rather than constituting a purely discretionary interpersonal exchange (Kim et al., 2008). Support seeking may therefore carry different social costs than in individualistic cultures, because asking for help may be perceived as burdening the group or as revealing inadequacy in ways that threaten face. Such dynamics may discourage explicit help seeking among victims of workplace bullying. At the same time, in-group solidarity norms may provide a stronger baseline of implicit or automatically available support that does not require direct solicitation. These considerations suggest that the mechanisms through which social support operates as a buffering resource may differ qualitatively from those documented in Western samples, even if the overall direction of the association between social support and work stress remains similar. The finding that the indirect effect through social support was statistically significant but modest (*β* = 0.024) may partly reflect cultural constraints on the mobilization and activation of support resources under conditions of workplace bullying.

Confucian heritage face saving dynamics may further amplify the psychological consequences of workplace bullying. In this cultural context, workplace bullying may threaten both personal well being and social standing, which may help explain the relatively strong direct association between workplace bullying and work stress after accounting for social support (*β* = 0.497) (Hwang, 1987; Hobfoll, 1989, 2001).

Taken together, these cultural considerations suggest that the present findings establish the empirical structure of the relationships among workplace bullying, work stress, and social support within East Asian organizational contexts, but they should not be assumed to generalize automatically across cultural settings. The Job Demands-Resources and Conservation of Resources frameworks provide theoretically coherent explanations of the observed patterns, yet the magnitude of the direct and indirect effects, and potentially the mechanisms through which these variables interact, are likely conditioned by cultural norms that vary across societies. Future research should therefore prioritize cross-cultural replication using measurement-equivalent instruments administered across societies that differ in power distance and individualism–collectivism. Such efforts would clarify whether the structural model observed in the present study is robust across cultural boundaries or is more specific to East Asian organizational contexts.

#### 5.2.2 Workplace bullying as a determinant of work stress

The moderate positive association between workplace bullying and work stress observed in the present meta-analysis (pooled r = 0.517) is consistent with and extends the findings of prior international meta-analytic reviews. Nielsen and Einarsen (2012) reported comparable effect sizes for the relationship between bullying and mental health outcomes, and Bowling and Beehr (2006) documented substantial associations between harassment and indicators of strain across diverse industry samples. Verkuil et al. (2015) demonstrated that these psychological associations are paralleled by physiological stress responses, providing triangulating evidence for the health impairment pathway posited by the JD-R model (Bakker and Demerouti, 2007). The present findings thus contribute to a convergent body of international evidence confirming that workplace bullying operates as a potent social stressor across cultural and occupational contexts.

Importantly, the present results extend beyond the nursing samples that have dominated much of the Taiwanese literature. Although healthcare settings remain a frequently studied context (Feng et al., 2020; Lee and Jung, 2020; Alshawush et al., 2022), the studies included in this meta-analysis also encompassed samples from the public sector (Liu et al., 2020), hospitality (Liu and Chou, 2016), education, and financial services (Chen et al., 2015), supporting the cross-occupational generalizability of the bullying-stress relationship. This aligns with Hauge et al. (2010), who demonstrated that work environment factors-rather than industry-specific characteristics-serve as the primary antecedents of workplace bullying. From the perspective of COR theory (Hobfoll, 1989, 2001), workplace bullying initiates resource loss spirals that are fundamentally similar across occupational contexts: the depletion of autonomy, interpersonal trust, and psychological safety constitutes a universal mechanism linking bullying exposure to elevated stress, irrespective of the specific industry setting.

#### 5.2.3 The erosion of social support under workplace bullying

The negative association between workplace bullying and social support is consistent with research demonstrating that bullied employees typically receive diminished support from colleagues and supervisors, resulting in lower well-being and reduced work efficiency (Warszewska-Makuch et al., 2015). When coworkers participate in or tolerate bullying behaviors, isolation tactics may be employed that heighten victims’ sense of exclusion; such dynamics often rely on personal knowledge not readily accessible to supervisors, allowing bullying to persist and damage organizational functioning (Han and Ha, 2016). In Taiwanese samples, nurses facing excessive workload and role stress reported that organizational resources, social support, and work enthusiasm served as protective factors against bullying (Su, 2017). Similarly, studies among civil servants indicated that positive emotions and social support alleviated the negative effects of bullying and moderated the link between bullying and burnout (Liu et al., 2020; Chen, 2018). Bullied employees may lose accumulated social resources over time; however, sufficient support can shape stress appraisals in more adaptive directions, reducing negative emotions and maladaptive coping strategies (Rossiter and Sochos, 2018). These convergent findings underscore that workplace bullying is associated with both direct psychological harm and diminished social resources that employees require to manage adversity.

#### 5.2.4 Social support as a buffer against work stress

The negative relationship between work stress and social support is consistent with the stress-buffering hypothesis, which posits that social support shapes individual adaptation under demanding work conditions (Wang et al., 2007; Yang, 2012). Prior research has suggested that social support may function as a moderator between work stressors and job performance, with employees who receive adequate support demonstrating greater capacity to manage stress and sustain productivity (Kao and Lu, 2011). Empirical evidence across diverse Taiwanese occupational groups supports this perspective: among medical employees, greater support was associated with reduced perceived stress (Chen et al., 2013; Yang et al., 2019); among civil servants, support buffered stress and improved coping outcomes (Yeh and Tseng, 2022; Huang et al., 2018); and in the financial sector, the degree of available support differentiated between employees who excelled under pressure and those whose performance deteriorated (Chen et al., 2015). These findings collectively reinforce the view that social support constitutes a critical psychosocial resource that mitigates the adverse effects of work.

#### 5.2.5 The partial mediating role of social support

The finding that social support partially mediates the relationship between workplace bullying and work stress is consistent with both the JD-R model and COR theory. Within the JD-R framework, workplace bullying represents a social job demand that initiates the health impairment process; social support, as a job resource, partially counteracts this process by reducing the perceived severity of demands and facilitating adaptive coping (Bakker and Demerouti, 2007; Bakker et al., 2005). The small but statistically significant magnitude of the indirect effect (*β* = 0.024, constituting 4.0% of the total effect) aligns with the JD-R proposition that the buffering capacity of any single resource is limited when demands are severe—as is typically the case with persistent bullying.

COR theory provides a complementary explanation for the predominance of the direct pathway. Because workplace bullying is associated with the erosion of the very interpersonal resources that constitute social support, victims experience a compounding resource loss that diminishes the availability and effectiveness of support as a coping mechanism (Hobfoll, 2001). This dynamic is consistent with the findings of Nielsen et al. (2020), who showed that social support’s protective function against bullying-related distress was attenuated in contexts where support resources were severely compromised. The present results thus suggest that while social support constitutes a statistically significant buffer, its protective capacity is constrained under conditions of sustained bullying—a pattern that underscores the importance of addressing bullying at its organizational roots rather than relying solely on support-based interventions.

These findings are further supported by O’Donnell and MacIntosh (2016), who demonstrated that bullied individuals typically cope by seeking social support from managers, colleagues, unions, or human resources departments, and by Yao (2012), who found that individuals with low levels of social support tend to experience more severe consequences of bullying. Hwang and Lin (2002) and Hwang (2010) reported analogous mediation patterns in which indirect pathways through social support were significant but modest in magnitude, consistent with the partial mediation observed in the present study. Samnani and Singh (2012) noted that social support operates at multiple levels—individual, group, and organizational—suggesting that the aggregate-level analysis in the present study may underestimate the total buffering effect, as support from different sources may operate through distinct mechanisms.

#### 5.2.6 Causality constraints and methodological considerations

Several methodological constraints bear directly on the interpretation of the present findings and must be explicitly acknowledged.

##### 5.2.6.1 Causality

The structural equation model tested in this study specifies directional paths among workplace bullying, work stress, and social support. However, these directional specifications are theoretically derived and cannot be confirmed as causal relationships on the basis of a correlational meta-analysis. The pooled correlation matrix used as input for the structural model was derived from predominantly cross-sectional primary studies, which do not provide information about temporal ordering. Consequently, the finding that workplace bullying is associated with work stress both directly and indirectly through social support should be interpreted as evidence of a correlational structure that is consistent with the proposed theoretical model—not as evidence of causal effects. Reverse causal pathways, in which elevated stress increases susceptibility to bullying or erodes the capacity to maintain supportive relationships, remain plausible and cannot be ruled out (Zapf et al., 2020). Reciprocal causation, in which bullying and stress mutually reinforce one another over time, is also consistent with longitudinal evidence from the broader occupational health literature (de Lange et al., 2004). The confirmation of the hypothesized structural model should therefore be regarded as providing theoretical plausibility rather than causal proof.

##### 5.2.6.2 Common method bias

Because all three constructs were measured exclusively through self-report instruments in the primary studies, the observed associations may be inflated by shared method variance (Podsakoff et al., 2003). The pooled effect sizes should accordingly be treated as upper-bound estimates. Future research employing multi-source designs, experience sampling methods, or objective indicators of stress (e.g., cortisol, absenteeism records) would provide more rigorous tests of the hypothesized relationships.

##### 5.2.6.3 Measurement heterogeneity

The high I^2^ values observed across all three meta-analyses (ranging from 94.00 to 97.29) indicate substantial heterogeneity in effect sizes that is not attributable to sampling error alone. This heterogeneity likely reflects, in part, the use of different measurement instruments, different operationalizations of the constructs, and different sample compositions across studies. While the random-effects model employed in this study accounts for between-study variability in the estimation of pooled effects and confidence intervals, it does not identify the sources of this variability. Moderator analyses examining the influence of measurement approach (e.g., NAQ vs. self-labeling for bullying), support dimension (e.g., supervisor vs. coworker support), and sample characteristics (e.g., occupation, cultural context) were not feasible given the limited number of studies in each pairwise analysis but represent an important direction for future research.

##### 5.2.6.4 Cultural generalizability

The restriction of the study sample to Taiwan and other Asian contexts limits the cultural generalizability of the findings. The magnitude and nature of the relationships among workplace bullying, work stress, and social support may differ across cultural settings that vary in power distance, collectivism, and organizational norms (Hofstede, 2001; Loh et al., 2010). The present findings should therefore be interpreted within the cultural context from which they were derived, and cross-cultural replication studies are needed to establish the boundary conditions of the observed associations.

#### 5.2.7 Measurement heterogeneity

The high I^2^ values observed across all three pairwise meta-analyses (94.00–97.29) indicate that the vast majority of between-study variability reflects true heterogeneity rather than sampling error. The descriptive analysis of measurement instruments (Table 1; Section IV.G) identifies instrument variation as a plausible contributor to this heterogeneity. Three specific sources of measurement-related variability warrant emphasis.

First, the use of behavioral experience inventories (NAQ-R) versus self-labeling methods for workplace bullying may produce systematically different effect sizes. Behavioral inventories assess exposure to specific negative acts and tend to yield higher prevalence estimates and stronger correlations with outcome variables, because they do not require respondents to cognitively appraise their experiences as constituting “bullying” (Nielsen et al., 2010; Einarsen et al., 2009). The pooled bullying-stress and bullying-support correlations in the present study therefore reflect a mixture of these measurement approaches, and the true variability in effect sizes may be partly methodological rather than substantive.

Second, the operationalization of work stress ranged from global perceived stress (PSS) to multidimensional role-based measures. These instruments capture partially different constructs: global perceived stress reflects an overall subjective appraisal, whereas role-specific measures index particular stressor domains. Correlations between bullying and role-specific stress may be inflated when the bullying behavior overlaps conceptually with the measured role stressor (e.g., task-related bullying and role overload), or attenuated when the stressor domain is distinct from the bullying experience.

Third, social support was operationalized at different levels of specificity, ranging from global aggregate scales to source-specific measures differentiating supervisor, coworker, and family support. This variation is theoretically important because the JD-R model identifies supervisor and coworker support as proximal job resources that buffer against workplace demands (Bakker et al., 2005), whereas family support operates through different mechanisms and may be less directly responsive to workplace bullying. Pooling across these distinct operationalizations may mask differential associations: supervisor support may show a stronger negative correlation with bullying (because supervisors are often the source of or witness to bullying behaviors), while family support may show a stronger buffering effect on stress (because it provides an external resource reservoir uncontaminated by workplace dynamics). The aggregate-level analysis in the present study cannot disaggregate these source-specific effects, representing a limitation that future research should address.

Taken together, these measurement-related considerations suggest that the pooled effect sizes should be interpreted as representing the average association across a heterogeneous set of operationalizations, and that the high I^2^ values are at least partly attributable to methodological variability rather than exclusively to substantive between-study differences. Moderator analyses examining instrument type as a source of heterogeneity were not feasible given the limited number of studies per subgroup (k = 2–5), but represent an important priority for future meta-analytic research as additional primary studies employing standardized instruments accumulate in this domain.

### 5.3 Practical and theoretical implications

#### 5.3.1 Implications for organizational practice

The empirical findings of the present study point to two tiers of organizational intervention, each aligned with a distinct pathway in the structural model.

##### 5.3.1.1 Primary interventions: reducing workplace bullying

The meta analytic results indicated a moderate positive association between workplace bullying and work stress. In the effect decomposition, the direct pathway accounted for approximately 96.1% of the total association (0.497/0.517), whereas the indirect pathway through social support accounted for approximately 3.9% (0.020/0.517). This pattern implies that the primary strategy for reducing work stress is to address workplace bullying at its source through clear anti bullying policies, equitable supervisory practices, and prompt institutional response mechanisms. At the same time, organizations should strengthen supportive resources because social support remains a meaningful, though secondary, pathway through which bullying relates to work stress (Chiu and Hsu, 2015; Wang et al., 2007; Kao and Lu, 2011).

##### 5.3.1.2 Complementary interventions: strengthening social support

The significant indirect pathway through social support (*β* = 0.024, *p* = 0.002), though small relative to the direct effect, confirms that social support plays a meaningful buffering role. Accordingly, organizations should invest in complementary support-enhancing strategies, including workplace counseling services, mental health education, and supervisor training in providing emotional and instrumental support. The negative association between work stress and social support (effect size = −0.201) further suggests that strengthening peer and supervisory support can reduce perceived work stress even when bullying is not the primary stressor (Wang et al., 2007; Kao and Lu, 2011). Promoting team cohesion and establishing two-way communication channels may further enhance employees’ access to supportive resources.

#### 5.3.2 Theoretical implications

This study contributes to the literature by offering a meta-analytically derived structural model that integrates findings across multiple empirical studies. Unlike prior research that has typically examined bivariate associations in isolation, the present study simultaneously assessed the direct and indirect pathways among workplace bullying, work stress, and social support, thereby providing a more comprehensive account of their interrelationships. The confirmation of partial mediation refines theoretical understanding by demonstrating that social support serves as a supplementary, rather than primary, mechanism through which bullying affects work stress. This finding suggests that theoretical models should account for the limited protective capacity of social support when bullying severity is high, and that alternative mediating or moderating variables may be necessary to fully explain the bullying-stress relationship.

### 5.4 Limitations

Several methodological constraints of the present study should be acknowledged.

First, the availability of correlation coefficients was limited by inconsistencies in how primary studies reported their statistical results. Although some included studies measured two or more of the focal variables, differences in analytical reporting conventions meant that pairwise correlation coefficients were not always provided. In certain cases, reported coefficients pertained to subdimensions rather than aggregate constructs, further reducing the pool of usable data for the meta-analysis.

Second, the literature search was confined to electronic databases—including the Airiti Library, Taiwan’s National Digital Library of Theses and Dissertations, Taiwan’s National Central Library of Periodical Literature, and Web of Science—and did not include print-only publications or theses and dissertations from countries outside Taiwan and Asia. This scope restriction may have excluded relevant empirical evidence that could have influenced the pooled effect sizes.

Third, variations in the operationalization of workplace bullying, work stress, and social support across the included studies introduced measurement heterogeneity. Differences in sample characteristics, survey instruments, and variable definitions were difficult to fully reconcile, as reflected in the high I^2^ values observed across all three meta-analyses, which may have affected the precision of the pooled estimates.

Fourth, the three constructs were examined at the aggregate level without differentiation into subdimensions. Given the multidimensional nature of workplace bullying (e.g., person-related vs. work-related bullying), work stress (e.g., role overload vs. role ambiguity), and social support (e.g., emotional vs. instrumental support), the aggregate-level analysis may have obscured heterogeneous patterns at the dimensional level.

Fifth, the study sample was geographically restricted to Taiwan and East Asia. This restriction was a deliberate scope decision motivated by the goal of pooling evidence from a culturally coherent region; however, it introduces boundary conditions that must be explicitly acknowledged. The cultural dimensions that characterize East Asian organizational environments—high power distance, collectivism, Confucian-heritage values, and face-saving norms—may systematically influence the prevalence and expression of workplace bullying, the availability and activation of social support, and the appraisal of work stress in ways that limit the transferability of the observed effect sizes and structural relationships to Western or other cultural contexts (Hofstede, 2001; Kim et al., 2008; Loh et al., 2010). The present findings should therefore be interpreted as establishing the correlational structure among these constructs within a specific cultural context rather than as confirming a culturally universal model. The direction of potential cultural bias is not uniform: power distance norms may attenuate bullying-stress correlations through underreporting, while face-related dynamics may amplify them through culturally specific stress appraisals. These competing influences cannot be disaggregated within the present design. Sixth, the present study relied exclusively on quantitative survey data synthesized through meta-analysis. The absence of qualitative evidence limits the depth of contextual understanding regarding the mechanisms through which workplace bullying, social support, and work stress interact in organizational settings.

Seventh, the structural equation model tested in this study is just-identified, with zero degrees of freedom. A just-identified model reproduces the observed correlation matrix exactly, precluding any test of overall model fit and preventing the comparison of the hypothesized model against alternative structural configurations (Kline, 2016). With only three constructs, no competing models with different path structures can be distinguished on the basis of fit. This is an inherent limitation of meta-analytic structural equation modeling when the number of constructs is small (Viswesvaran and Ones, 1995; Cheung and Chan, 2005). The interpretive weight of the structural model therefore rests on the theoretical plausibility of the specified paths and the statistical properties of the individual parameter estimates, rather than on evidence of superior model fit relative to alternatives. Future research incorporating additional constructs—such as organizational climate, coping strategies, or individual difference variables—would permit the specification of overidentified models in which global fit indices carry diagnostic value and alternative causal configurations can be formally tested.

Eighth, the number of primary studies contributing to each pairwise meta-analysis was relatively small (k = 9 to 11). Small study pools limit the precision of pooled effect size estimates, reduce statistical power for detecting moderator effects and publication bias, and may compromise the stability of path coefficients derived from meta-analytic structural equation modeling. Although the leave-one-out sensitivity analysis confirmed that no single study exerted a disproportionate influence on the pooled estimates, the results of the structural model should be interpreted with caution given the limited number of contributing studies. The bootstrap procedure used to test the indirect effect provides a non-parametric assessment of significance that is less dependent on large-sample assumptions; nevertheless, replication with a larger and more diverse evidence base is warranted as additional primary studies accumulate in this domain (Cheung and Chan, 2005; Viswesvaran and Ones, 1995).

### 5.5 Suggestions for future research

#### 5.5.1 Measurement heterogeneity

The constructs of workplace bullying, work stress, and social support are each operationalized through multiple instruments that differ in content, dimensionality, and response format. Workplace bullying is measured by behavioral experience inventories (e.g., the Negative Acts Questionnaire; Einarsen et al., 2009), self-labeling approaches, or composite measures combining both methods. These approaches have been shown to yield different prevalence estimates and effect sizes (Nielsen et al., 2010). Work stress is variously assessed as perceived stress, role-based stress (role conflict, role ambiguity, role overload), emotional exhaustion, or composite strain indices. Social support is measured in terms of perceived availability, received support, or source-specific support (supervisor, coworker, family), with each dimension potentially operating through distinct mechanisms (Haber et al., 2007). This measurement heterogeneity complicates the interpretation of pooled effect sizes in meta-analyses, as correlations derived from different operationalizations may reflect partially different constructs. Although meta-analytic pooling provides an overall estimate, it may mask meaningful variation attributable to instrument selection rather than to substantive differences in the underlying relationships.

#### 5.5.2 Cross-sectional dominance and causal inference

The overwhelming reliance on cross-sectional survey designs in the workplace bullying literature represents a fundamental constraint on causal interpretation. Cross-sectional data capture associations at a single point in time and cannot establish the temporal ordering of variables necessary for causal claims (Maxwell and Cole, 2007). The structural equation model tested in the present study specifies directional paths from workplace bullying to work stress and social support, and from social support to work stress. Although these paths are theoretically motivated, the cross-sectional nature of the primary studies means that the meta-analytically derived correlation matrix does not contain information about temporal precedence. Consequently, the structural model should be understood as a test of whether the observed correlational structure is consistent with the hypothesized causal ordering, rather than as evidence that workplace bullying causes work stress or that social support causally mediates this relationship. Alternative causal configurations—including reverse causation (stress increases vulnerability to bullying) and reciprocal causation (bullying and stress mutually reinforce one another)—remain empirically viable and cannot be ruled out by the present design (Zapf et al., 2020). The limited number of longitudinal studies in this domain constitutes a significant gap that constrains the strength of causal inferences that can be drawn from the accumulated evidence.

#### 5.5.3 Common method bias

A further methodological concern is the pervasive reliance on self-report questionnaire data across the studies reviewed. When all variables in a study are measured through self-report instruments administered to the same respondents at the same time, the resulting correlations may be inflated by common method variance (Podsakoff et al., 2003). Shared response tendencies—including acquiescence bias, social desirability, and negative affectivity—can produce artifactual covariation among constructs that are in reality less strongly related than the observed coefficients suggest. Spector (2006) has argued that negative affectivity, in particular, may simultaneously inflate self-reports of workplace stressors, strain, and interpersonal difficulties, thereby confounding the relationships among bullying, stress, and support. None of the primary studies included in the present meta-analysis employed multi-source designs, objective indicators, or latent method factor corrections that would mitigate this concern. The pooled effect sizes reported in this study should therefore be interpreted as upper-bound estimates that may partly reflect shared method variance.

#### 5.5.4 Cultural and contextual considerations

The primary studies included in this meta-analysis are drawn predominantly from Taiwan and other East Asian societies, which are characterized by relatively high levels of collectivism, power distance, and hierarchical organizational structures (Hofstede, 2001). These cultural dimensions may influence the prevalence, expression, perception, and consequences of workplace bullying in ways that limit the cross-cultural generalizability of the findings. In high power-distance cultures, employees may be more reluctant to report bullying by supervisors, potentially leading to underestimation of bullying prevalence and attenuation of observed associations (Loh et al., 2010). Collectivist norms may also shape the nature and effectiveness of social support: support that is embedded in obligation and hierarchy may function differently from support exchanged among peers in individualistic settings (Kim et al., 2008). Furthermore, the concept of “work stress” may carry different connotations across cultural contexts, with some societies normalizing high workload as a professional expectation rather than a source of strain. These cultural dynamics are rarely examined as moderators in the reviewed studies, representing an important limitation of the evidence base.

#### 5.5.5 Occupational sample representativeness

The empirical literature on workplace bullying, work stress, and social support has been disproportionately concentrated in healthcare settings, particularly among nursing populations (Alshawush et al., 2022; Feng et al., 2020; Lee and Jung, 2020). While healthcare represents a high-risk context for bullying due to hierarchical structures, emotional demands, and staffing pressures, the over-representation of nursing samples raises questions about the extent to which observed effect sizes generalize to other industries. Occupational contexts differ in power structures, role clarity, team composition, and the availability of formal support mechanisms, all of which may moderate the relationships among bullying, stress, and support. The inclusion of samples from hospitality (Liu and Chou, 2016), the public sector (Liu et al., 2020), financial services (Chen et al., 2015), and education (Fahie, 2014) partially addresses this concern, but the overall evidence base remains skewed toward healthcare. Future research should prioritize under-studied sectors—including manufacturing, technology, and the gig economy—to establish whether the observed relationships are robust across the full range of contemporary work environments.

## 6 Conclusion

This study provides a meta analytically grounded account of the relationships among workplace bullying, work stress, and social support. Workplace bullying was positively associated with work stress and negatively associated with social support, and social support was negatively associated with work stress. In the observed variable path analysis based on the pooled correlation matrix, the indirect pathway through social support was small (*β* = 0.020) relative to the direct pathway from workplace bullying to work stress (*β* = 0.497), yielding a total effect of *β* = 0.517. These findings indicate that social support functions as a supplementary pathway in the broader association between workplace bullying and work stress, while the direct association remains the predominant pattern.

## Data Availability

The raw data supporting the conclusions of this article will be made available by the authors, without undue reservation.
