# Supplementary material for: Correlations among workplace bullying, work stress, and social support: a meta-analysis with structural equation modeling
Source: Front Psychol. 2026 May 8;17:1817200. doi: 10.3389/fpsyg.2026.1817200 (PMC13193934; doi:10.3389/fpsyg.2026.1817200)
Supplement: Supplementary file 1 [file Supplementary_file_1.docx]

# Appendix I: PRISMA 2020 Checklist

| **Section** | **#** | **Checklist Item** | **Location** |
| --- | --- | --- | --- |
| Title | 1 | Identify the report as a systematic review | Title |
| Abstract | 2 | Structured summary | Abstract |
| Rationale | 3 | Rationale for the review | Section I |
| Objectives | 4 | Objectives/questions | Section I, Hypotheses 1-4 |
| Eligibility | 5 | Inclusion/exclusion criteria | Section III.A |
| Info sources | 6 | Databases and date of last search | Section III.B |
| Search strategy | 7 | Full search strategies | Section III.B |
| Selection | 8 | Selection process and reviewers | Section III.C |
| Data collection | 9 | Data collection process | Section III.D |
| Data items | 10a | Outcomes sought | Section III.A (PECOS) |
| Data items | 10b | Other variables | Section III.D |
| Risk of bias | 11 | Risk of bias assessment | Section III.A, Criterion 5 |
| Effect measures | 12 | Effect measures used | Section III.E (Data Analysis) |
| Synthesis | 13a-f | Synthesis methods, heterogeneity, sensitivity | Section III.E-F |
| Reporting bias | 14 | Publication bias assessment | Section IV (Fail-safe N) |
| Study selection | 16a | Search and selection results with flow diagram | Section III.C; Figure 1. |
| Study chars. | 17 | Characteristics of included studies | Section III.D; coding database |
| Results | 19-20 | Individual study and synthesis results | Section IV, Tables 1-5 |
| Reporting biases | 21 | Publication bias results | Section IV, Table 2 |
| Discussion | 23a-d | Interpretation, limitations, implications | Section V.A-E |
| Registration | 24a | Registration status | Section III.E |
| Funding | 26 | Funding sources | Declarations |
| Conflicts | 27 | Competing interests | Declarations |

*Note.* This checklist follows the PRISMA 2020 statement (Page et al., 2021). Items not applicable to the present correlational meta-analysis (e.g., Items 15, 22) are omitted.

# Appendix II: Measurement Instruments Used in Included Studies

| **Construct** | **Instrument** | **k** | **Characteristics** |
| --- | --- | --- | --- |
| Workplace bullying | Negative Acts Questionnaire-Revised (NAQ-R; Einarsen et al., 2009) | 5 | 22-item behavioral experience inventory; person-related, work-related, physically intimidating subscales |
|  | Self-labeling single item or adapted scales | 3 | Self-classification as bullying victim; may yield lower prevalence and weaker correlations (Nielsen et al., 2010) |
|  | Researcher-developed or locally adapted scales | 4 | Chinese-language instruments adapted to Taiwanese occupational contexts; variable item counts and factor structures |
| Work stress | Perceived Stress Scale (PSS; Cohen et al., 1983) | 3 | Global perceived stress; 10- or 14-item versions |
|  | Role-based stress measures (role conflict, role ambiguity, role overload) | 4 | Multidimensional; captures specific stressor types rather than global strain |
|  | Job Stress Scale or adapted occupational stress scales | 5 | Occupation-specific; variable dimensional structures across studies |
| Social support | Multidimensional Scale of Perceived Social Support (MSPSS; Zimet et al., 1988) | 4 | 12-item; family, friends, significant other subscales |
|  | Supervisor/coworker support scales (e.g., from JCQ; Karasek et al., 1998) | 3 | Source-specific workplace support; distinguishes supervisor from coworker |
|  | Global or adapted social support scales | 5 | Unidimensional or locally developed; collapses support sources into aggregate score |

*Note.* k = number of included studies using each instrument type. Some studies used multiple instruments; instrument counts may exceed the total number of studies for a given pairwise analysis. NAQ-R = Negative Acts Questionnaire-Revised; PSS = Perceived Stress Scale; MSPSS = Multidimensional Scale of Perceived Social Support; JCQ = Job Content Questionnaire.
